# Enhancing sepsis management: the role of dynamic heparin-binding protein monitoring in elderly patients

**DOI:** 10.3389/fmed.2025.1538515

**Published:** 2025-06-11

**Authors:** Lei Miao, Dandan Liu, Jingxian Liao, Xiaozhu Shen, Chunhui Xie, Guangyun Hu

**Affiliations:** ^1^Department of Critical Care Medicine, The Second People’s Hospital of Lianyungang, Lianyungang, China; ^2^Department of Geriatrics, The Second People’s Hospital of Lianyungang, Lianyungang, China

**Keywords:** elderly, sepsis, heparin-binding protein, dynamic monitoring, prognosis

## Abstract

**Background:**

Sepsis is a life-threatening condition with particularly high mortality rates among the elderly. This study investigates the dynamic monitoring of heparin-binding protein (HBP) levels as prognostic biomarkers to improve risk stratification and management in elderly septic patients.

**Methods:**

We conducted a retrospective cohort study involving patients aged 65 and older who were hospitalized for sepsis. Data were extracted from electronic medical records, including demographic, clinical, and laboratory information. We analyzed the relationship between dynamic HBP levels and 28-day mortality using linear mixed-effects models to assess the effects of time and prognostic groups.

**Results:**

Among 386 elderly septic patients, the 28-day mortality rate was 20.73%. HBP levels were significantly elevated at all time points in the mortality group compared to the survival group (*p* < 0.001). The linear mixed-effects model indicated that time, prognosis group, and their interaction significantly influenced HBP levels. In the survival group, HBP levels decreased significantly over time, whereas the mortality group exhibited a smaller reduction, with HBP levels remaining elevated overall. The prognostic predictive ability of HBP improved at various time points, with the combined model of HBP and C-reactive protein (CRP) showing time-varying area under the curve (AUC) values: 0.728 on day 10, 0.744 on day 15, and 0.803 on day 20. The time-dependent ROC curve demonstrated that the combined model consistently exhibited superior discriminative ability throughout the follow-up period. Additionally, the time-dependent Cox regression model indicated that dynamic HBP levels effectively predicted 28-day mortality risk across all subgroups (*p* < 0.001).

**Conclusion:**

Dynamic monitoring of HBP levels may aid in risk stratification and support clinical decision-making. Further prospective studies are required to evaluate its clinical utility and potential impact on patient management.

## Introduction

Sepsis is a complex syndrome characterized by a dysregulated host response to infection, leading to systemic inflammation, organ dysfunction, and, ultimately, increased mortality (1). It is particularly prevalent among elderly patients, who often present with atypical symptoms and have multiple comorbidities that complicate diagnosis and treatment (2). The World Health Organization (WHO) has identified sepsis as a global health priority, emphasizing the need for improved diagnostic and prognostic tools to enhance patient outcomes (3). In this context, identifying reliable biomarkers that can predict sepsis prognosis is crucial for optimizing management strategies and improving survival rates (4).

Among the various biomarkers investigated in sepsis, heparin-binding protein (HBP) has emerged as a promising candidate. HBP is a multifunctional inflammatory mediator stored in neutrophil granules and released rapidly in response to infection and endothelial activation (5, 6). Elevated HBP levels are considered indicators of systemic inflammatory response and microvascular dysfunction, both closely linked to the severity and mortality risk in sepsis (7, 8). HBP has demonstrated significant sensitivity and specificity as a biomarker for the early diagnosis and prognosis of sepsis in several studies (9).

However, interpreting HBP levels in elderly patients presents unique challenges. Aging is associated with immunosenescence and a persistent low-grade inflammatory state (“inflammaging”), which can affect both the baseline levels and the magnitude of increase of inflammatory biomarkers such as HBP (10). Furthermore, the high prevalence of chronic comorbidities and endothelial dysfunction in elderly patients may further influence HBP kinetics and its release upon infectious or inflammatory stimulation (11). These age-related physiological changes may impact the diagnostic and prognostic performance of HBP specifically in the elderly, making it crucial to investigate its clinical utility in this population.

Additionally, elderly patients with sepsis frequently experience distinct clinical features and worse outcomes compared to younger individuals, due to age-related decline in immune function, multiple comorbidities, and altered pharmacokinetics (12, 13). This leads to a higher incidence of severe infections and increased risk of adverse outcomes (14), underscoring the need for effective prognostic tools tailored to this demographic.

While previous research has demonstrated that static HBP measurements can predict mortality in sepsis (15), the dynamic trends of HBP over time—especially in elderly patients—have not been thoroughly elucidated. Given these physiological and clinical considerations, we hypothesized that the prognostic value and temporal patterns of HBP in elderly sepsis patients might differ from that in younger populations. Therefore, the present study aims to analyze the dynamic changes in HBP levels in elderly patients with sepsis and evaluate their prognostic significance.

By investigating the relationship between dynamic HBP trends and 28-day mortality, as well as the combined predictive value of HBP and C-reactive protein (CRP), this study seeks to provide new insights to enhance clinical risk stratification and management for this vulnerable population. Ultimately, we hope our findings will inform more targeted and effective interventions, contributing to improved survival rates and quality of life for elderly patients with sepsis.

## Methods

### Study design and patients

This retrospective cohort study adhered to the STROBE cohort reporting guidelines. Patients aged 65 years or older who were hospitalized for sepsis at the Second People’s Hospital of Lianyungang between May 1, 2020, and May 1, 2024, were included. Lianyungang Second People’s Hospital is a tertiary (Grade III, Class A) comprehensive teaching hospital located in Lianyungang, Jiangsu Province, China, and serves as a major regional referral center providing a wide range of medical and surgical services to a diverse patient population. Follow-up for mortality due to sepsis was conducted within 28 days post-discharge, primarily using data from the hospital’s medical records system.

### Inclusion criteria

1. Diagnosis of sepsis according to the “Sepsis-3” criteria (i.e., organ dysfunction due to infection, with a SOFA score increase of ≥2 points) (1).
2. Age ≥ 65 years.
3. Availability of complete clinical data records, including dynamic monitoring of inflammatory factors such as HBP and CRP.
4. Hospitalization duration of ≥3 days (to ensure the availability of dynamic data).

### Exclusion criteria

1. End-stage disease states (e.g., advanced malignant tumors, end-stage liver disease).
2. Death within 48 h of hospitalization without dynamic monitoring data.
3. Coexisting severe immunosuppressive diseases (e.g., AIDS, long-term use of immunosuppressants).
4. Significant missing data (e.g., missing HBP or CRP data at critical time points).

The study received approval from the Ethics Committee of the Second People’s Hospital of Lianyungang (No. 2024KY094) and adhered to the ethical principles established by the Declaration of Helsinki. Given the retrospective nature of the study, no direct intervention in the clinical diagnosis or treatment of patients was conducted; the focus was solely on pre-existing clinical data. Consequently, the requirement for informed consent was waived by the Ethics Committee.

### Data collection

Clinical data were collected by trained researchers utilizing the electronic medical record system of the Second People’s Hospital of Lianyungang. The data collection encompassed patients’ demographic information, clinical characteristics, laboratory test results, and treatment measures. Specific data collected included:

Demographic information: Gender, age.

Clinical characteristics: Body Mass Index (BMI), Charlson Comorbidity Index (CCI) for assessing and documenting comorbidities, and infection sites.

Disease severity scores: APACHE II score—assessment and documentation of patients’ Acute Physiology and Chronic Health Evaluation. SOFA score—assessment and documentation of patients’ Sequential Organ Failure Assessment score.

Inflammatory factors: Heparin-binding protein and C-reactive protein measured on the day of admission (T0), 24 h after admission (T1), and on the third day after admission (T2).

Laboratory test results: Blood lactate levels (Lac), albumin (Alb), Aspartate Aminotransferase (AST), Alanine Aminotransferase (ALT), white blood cell count (WBC), platelet count (PLT), hemoglobin (HB), blood urea nitrogen (BUN), glucose (Glu).

Baseline vital signs, including blood pressure, heart rate, respiratory rate, and temperature, were defined as the first documented measurements upon hospital admission and, when feasible, should be collected before the initiation of vasopressor therapy. Patient outcomes, specifically survival or death, were documented following a short-term follow-up period of 28 days.

### Body mass index calculation

BMI is calculated using the formula: BMI = weight in kilograms/ (height in meters)^2^. It is a widely used measure to categorize individuals based on their body weight relative to height, providing insights into nutritional status and potential health risks (16).

### Charlson comorbidity index

The Charlson Comorbidity Index (CCI) is a method for predicting mortality by classifying and weighting comorbidities. Each comorbidity is assigned a score based on its potential impact on mortality, and the total score is the sum of all individual scores. A higher CCI indicates a greater burden of comorbid conditions and an increased risk of mortality (17).

### APACHE II scoring method

The Acute Physiology and Chronic Health Evaluation II (APACHE II) score assesses the severity of disease in critically ill patients. It incorporates physiological measurements, age, and chronic health conditions. The score ranges from 0 to 71, with higher scores indicating more severe illness and a higher risk of mortality (18).

### SOFA scoring method

The Sequential Organ Failure Assessment (SOFA) score evaluates the extent of a patient’s organ function or rate of failure. It considers six organ systems: respiratory, cardiovascular, hepatic, coagulation, renal, and neurological. Each system is scored from 0 to 4, with higher scores indicating greater organ dysfunction (19).

### Statistical analysis

Statistical analyses were performed using IBM SPSS Statistics version 21 and R version 4.4.1. Multiple imputation was employed to handle random missing values; for systematic missingness (such as data loss at later time points due to early death), a competing risks model was utilized in the analysis. Descriptive statistics were computed for all variables to summarize data characteristics. Continuous data that followed a normal distribution were analyzed using the Student’s t-test, with results reported as mean ± standard deviation (SD). Conversely, continuous variables not conforming to a normal distribution were analyzed using the non-parametric Mann–Whitney U test, with results expressed as median (interquartile range) [M (P25, P75)]. For categorical variables, the Chi-squared test was employed to compare group differences and assess associations among categorical data.

The dynamic changes in C-reactive protein (CRP) and heparin-binding protein (HBP) levels were analyzed using linear mixed-effects models to assess the effects of time, prognostic group, and their interaction on CRP and HBP levels. The main effects of time and prognosis were evaluated, and interactions were tested to determine differences in biomarker levels between the groups at different time points (T0, T1, T2). Receiver Operating Characteristic (ROC) curve analysis was conducted to evaluate the predictive performance of HBP, CRP, and their combined model for 28-day mortality. Time-varying area under the curve (AUC) values for HBP, CRP, and the combined model were calculated at different time points: day 10, day 15, and day 20, with time-varying ROC curves plotted to assess the classification ability of dynamic HBP. The correlation between the dynamic levels of HBP and the 28-day mortality risk of patients was analyzed using a time-dependent Cox regression model. The results were further analyzed in detail by subgroups based on gender, age, hypertension, diabetes, and infection sites. All statistical tests were two-sided, with a significance level set at *p* < 0.05.

## Results

### Clinical characteristics among different groups

**Figure 1:**
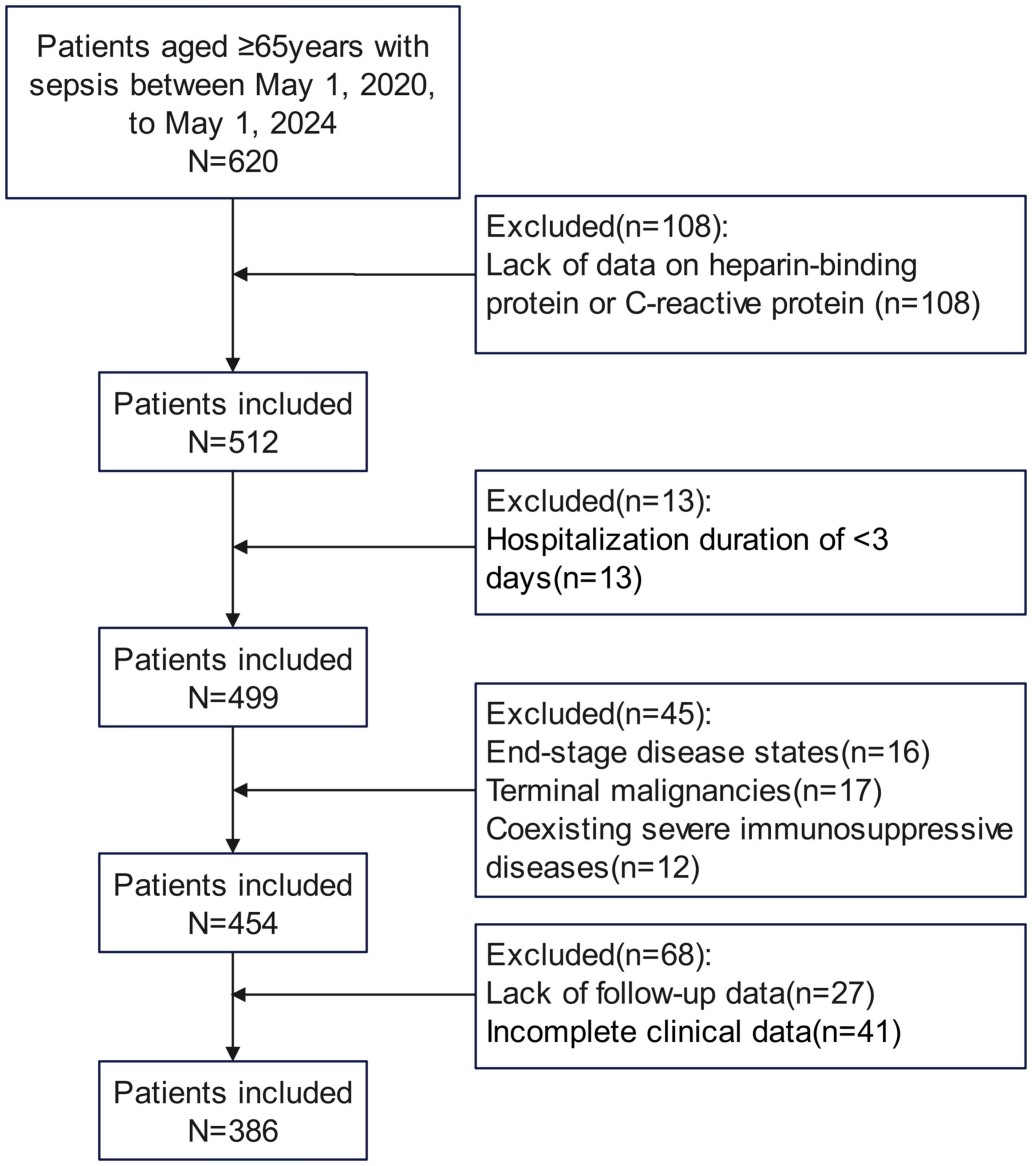
Flow diagram displaying the progress of all participants through the study.

A total of 386 elderly patients diagnosed with sepsis were enrolled in the study, adhering to specific inclusion and exclusion criteria, as shown in Figure 1. The 28-day mortality rate was 20.73%. As presented in Table 1, the average age of the death group (81.29 ± 5.27 years) was significantly higher than that of the survival group (77.11 ± 6.60 years, *p* < 0.001). Additionally, the APACHE II score was significantly elevated in the death group (18.61 ± 4.12) compared to the survival group (15.31 ± 4.10, *p* < 0.001). The SOFA score in the death group (7.40 ± 1.51) was also significantly higher than that in the survival group (5.95 ± 1.44, *p* < 0.001). Furthermore, the Charlson Comorbidity Index (CCI) indicated that the death group’s CCI (3.89 ± 1.06) was significantly greater than that of the survival group (2.94 ± 0.93, *p* < 0.001). At time points T0, T1, and T2, C-reactive protein levels were higher in the death group than in the survival group (*p* < 0.05). Heparin-binding protein (HBP) levels at all time points were also significantly elevated in the death group compared to the survival group (*p* < 0.001). Moreover, blood lactate levels in the death group were significantly higher, while serum albumin levels were significantly lower than those in the survival group (*p* < 0.001). No significant differences were observed between the two groups for other variables such as gender, BMI, underlying diseases, and infection sites (*p* > 0.05).

**Table 1:** Clinical characteristics among different groups.

Variables	Total (*n* = 386)	Survival group (*n* = 306)	Death group (*n* = 80)	*p*-value
Gender				0.259
Male, *n* (%)	197 (51.0)	161 (52.6)	36 (45.0)	
Female, *n* (%)	189 (49.0)	145 (47.4)	44 (55.0)	
Age, mean (SD)	77.98 (6.56)	77.11 (6.60)	81.29 (5.27)	<0.001
BMI, kg/m^2^, mean (SD)	22.92 (2.74)	23.05 (2.67)	22.40 (2.95)	0.076
Underlying diseases				
Hypertension, *n* (%)	150 (38.9)	120 (39.2)	30 (37.5)	0.798
Diabetes, *n* (%)	138 (35.8)	103 (33.7)	35 (43.8)	0.116
Cardiovascular disease, *n* (%)	98 (25.4)	75 (24.5)	23 (28.8)	0.471
COPD, *n* (%)	109 (28.2)	86 (28.1)	23 (28.8)	0.890
Cerebrovascular disease, *n* (%)	133 (34.5)	103 (33.7)	30 (37.5)	0.512
Infection site				0.724
Pulmonary, *n* (%)	104 (26.9)	82 (26.8)	22 (27.5)	
Abdominal, *n* (%)	94 (24.4)	71 (23.2)	23 (28.8)	
Urinary, *n* (%)	123 (31.9)	100 (32.7)	23 (28.8)	
Other, *n* (%)	65 (16.8)	53 (17.3)	12 (15.0)	
APACHII, mean (SD)	15.99 (4.31)	15.31 (4.10)	18.61 (4.12)	<0.001
SOFA, mean (SD)	6.25 (1.57)	5.95 (1.44)	7.40 (1.51)	<0.001
CCI, mean (SD)	3.14 (1.03)	2.94 (0.93)	3.89 (1.06)	<0.001
HR, beats/min, mean (SD)	85.35 (17.02)	85.47 (17.55)	84.91 (14.91)	0.796
SBP, mmHg, mean (SD)	125.16 (25.76)	126.91 (26.51)	118.45 (21.51)	0.009
DBP, mmHg, mean (SD)	71.07 (12.75)	71.42 (12.85)	69.71 (12.37)	0.287
CRP T0, mg/L, median (IQR)	55.85 (36.68, 75.98)	53.70 (35.38, 73.48)	65.95 (42.40, 79.40)	0.003
CRP T1, mg/L, median (IQR)	63.29 (46.68, 79.80)	60.08 (45.28, 77.14)	77.25 (54.65, 89.31)	<0.001
CRP T2, mg/L, median (IQR)	43.30 (32.20, 66.23)	41.10 (31.18, 56.60)	66.35 (45.50, 88.78)	<0.001
HBP T0, ng/mL, median (IQR)	46.86 (30.68, 81.73)	40.24 (27.65, 74.86)	80.83 (44.05, 121.07)	<0.001
HBP T1, ng/mL, median (IQR)	42.10 (28.80, 76.90)	34.95 (26.40, 59.38)	80.01 (54.40, 117.83)	<0.001
HBP T2, ng/mL, median (IQR)	26.50 (18.65, 45.50)	22.40 (17.50, 32.63)	76.91 (45.35, 109.04)	<0.001
White blood cell, ×109/L, mean (SD)	13.15 (4.06)	12.97 (3.90)	13.83 (4.59)	0.089
Platelet, ×109/L, mean (SD)	175.32 (81.39)	178.09 (85.42)	164.73 (62.99)	0.191
Hemoglobin, g/L, mean (SD)	101.61 (22.43)	101.02 (23.43)	103.86 (18.07)	0.314
Lactate, mmol/L, median (IQR)	3.46 (1.93)	3.15 (1.68)	4.62 (2.33)	<0.001
Albumin, g/L, mean (SD)	32.88 (3.95)	33.42 (3.94)	30.82 (3.27)	<0.001
Aspartate Aminotransferase, U/L, median (IQR)	32.00 (25.00, 44.25)	32.00 (25.00, 43.00)	33.00 (25.00, 47.75)	0.323
Alanine Aminotransferase, U/L, median (IQR)	31.00 (21.00, 42.00)	31.50 (21.75, 42.00)	29.00 (20.00, 43.00)	0.966
Blood urea nitrogen, mg/dL, mean (SD)	22.16 (6.73)	21.58 (6.71)	24.39 (6.41)	0.001
Glucose, mmol/L, mean (SD)	9.25 (3.51)	9.03 (3.34)	10.13 (4.03)	0.013

BMI, Body Mass Index; COPD, Chronic Obstructive Pulmonary Disease; APACHE II, Acute Physiology and Chronic Health Evaluation II; SOFA, Sequential Organ Failure Assessment; CCI, Charlson Comorbidity Index; HR, Heart Rate; SBP, Systolic Blood Pressure; DBP, Diastolic Blood Pressure; CRP, C-reactive protein; HBP, Heparin-binding protein; IQR, Interquartile Range; SD, standard deviation.

### Dynamic changes in CRP levels and their relationship with prognosis

We analyzed the dynamic changes in C-reactive protein (CRP) levels at different time points (T0, T1, T2) among patients in different prognostic groups. A linear mixed-effects model was employed to assess the effects of time, prognostic group, and their interaction on CRP levels.

Main effect of time: Compared to the baseline time point T0, CRP levels in the survival group significantly increased at T1 (β = 8.183, 95% CI [4.569, 11.797], *p* < 0.001) and significantly decreased at T2 (β = −9.876, 95% CI [−13.490, −6.262], *p* < 0.001). This indicates that, over time, the inflammatory levels in the survival group initially rise and then decline.

Main effect of prognosis: Overall CRP levels in the death group were significantly higher than those in the survival group (β = 10.383, 95% CI [3.474, 17.292], *p* = 0.003). At baseline, CRP levels in the death group were already significantly elevated compared to the survival group, suggesting that patients with poor prognosis had higher inflammatory levels upon admission.

**Figure 2:**
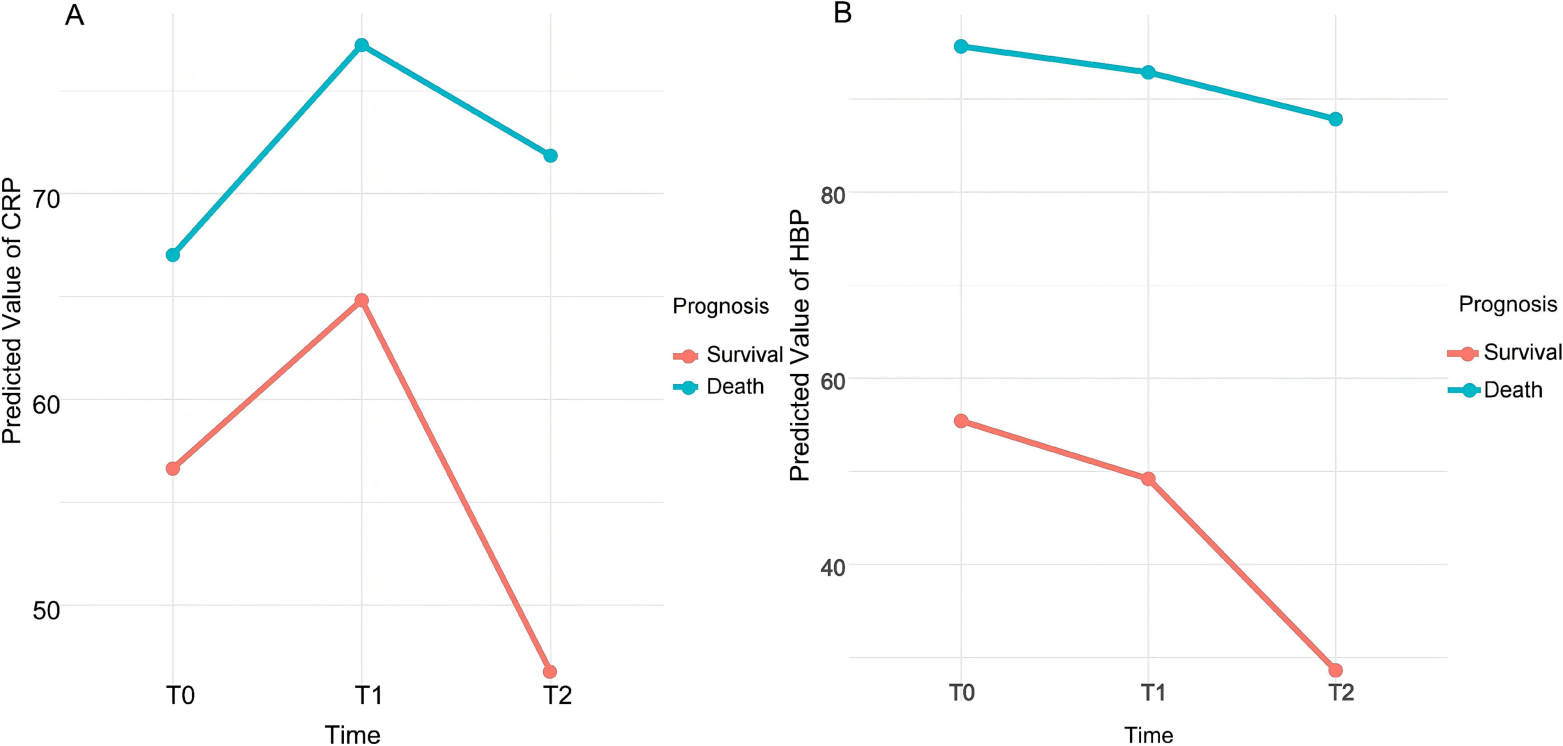
Dynamic trend of predicted values for CRP **(A)** and HBP **(B)**. CRP: C-reactive protein; HBP: Heparin-binding protein; T0: On the day of admission; T1: 24 h after admission; T2: On the third day after admission.

Interaction between time and prognostic group: At T1, the interaction between time and prognostic group was not significant (β = 2.020, *p* = 0.618), indicating no significant difference in CRP level changes between the death and survival groups at this time point. However, at T2, a significant interaction was observed (β = 14.699, 95% CI [6.753, 22.645], *p* < 0.001), suggesting that the trend of CRP level changes at T2 differed significantly between the death and survival groups. Figure 2A illustrates the changes in CRP levels at each time point for patients in different prognostic groups.

### Dynamic changes in HBP levels and their relationship with prognosis

We analyzed the dynamic changes in heparin-binding protein (HBP) levels at different time points (T0, T1, T2) among patients in various prognostic groups. A linear mixed-effects model was employed to assess the effects of time, prognostic group, and their interaction on HBP levels.

Main effect of time: In the survival group, HBP levels significantly decreased over time. Compared to the baseline time point T0, HBP levels at T1 showed a significant decline (β = −6.225, 95% CI [−10.244, −2.206], *p* = 0.0024). At T2, HBP levels further decreased significantly (β = −26.801, 95% CI [−30.820, −22.782], *p* < 0.001).

Main effect of prognosis: Overall HBP levels in the death group were significantly higher than those in the survival group (β = 40.229, 95% CI [30.540, 49.918], *p* < 0.001). This indicates that patients with poor prognosis had higher HBP levels at all time points.

Interaction between time and prognostic group: At T1, the interaction between time and prognostic group was not significant (β = 3.442, *p* = 0.4435), indicating no significant difference in HBP level changes between the death and survival groups at this time point. However, at T2, a significant interaction was observed (β = 18.999, 95% CI [10.178, 27.820], *p* < 0.001), suggesting that the patterns of HBP level changes over time differed between the prognostic groups. Figure 2B illustrates the trends in HBP levels at each time point for patients in the survival and death groups.

### ROC curves for HBP, CRP, and combined testing in prognostic prediction

**Figure 3:**
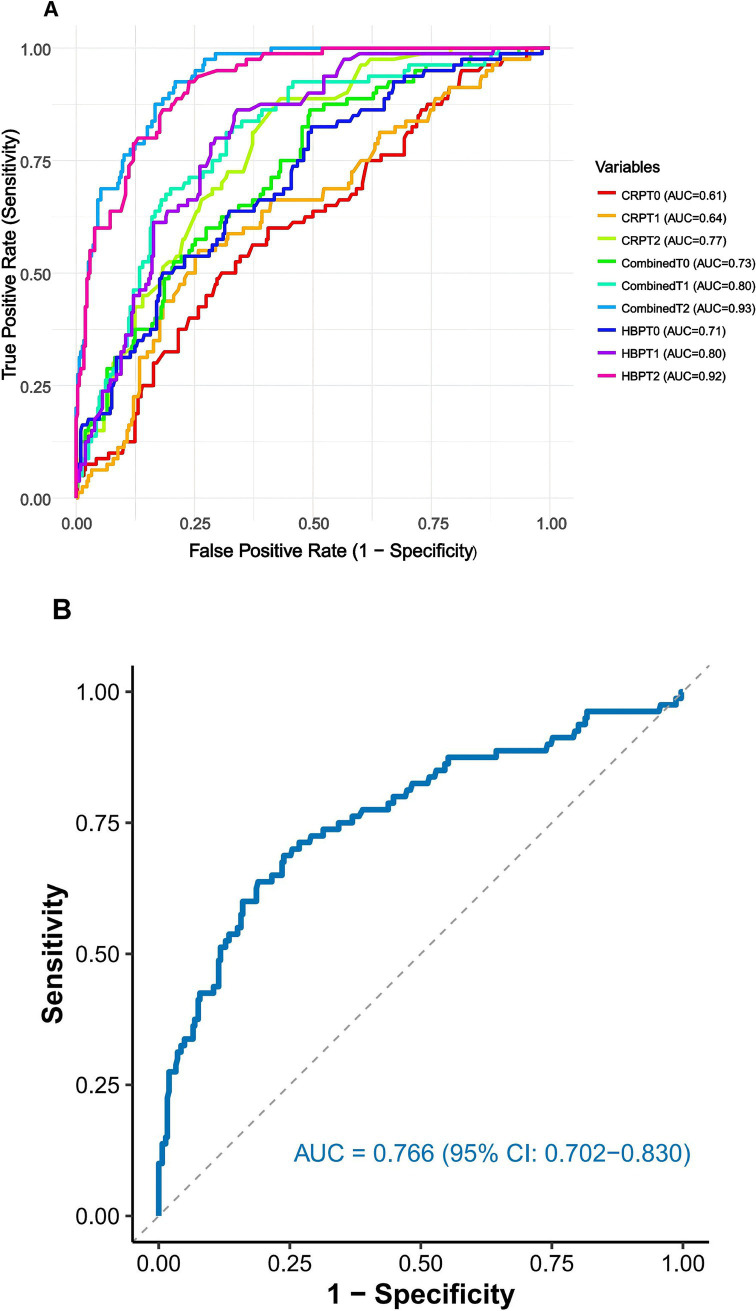
**(A)** ROC curves for HBP, CRP, and combined testing in prognostic prediction. **(B)** ROC curve for the relative change in HBP from baseline to Day 2 for predicting 28-day mortality. The relative change was calculated as [(HBP T2 – HBP T0)/HBP T0] × 100%. CRP: C-reactive protein; HBP: Heparin-binding protein; AUC: Area under the curve; T0: On the day of admission; T1: 24 h after admission; T2: On the third day after admission.

We evaluated the prognostic predictive capabilities of heparin-binding protein (HBP) and C-reactive protein (CRP) at different time points in elderly patients with sepsis. The results presented in Figure 3A indicate that at time point T0, HBP showed moderate predictive ability with an AUC of 0.710 (95% CI: 0.648–0.772). The optimal cut-off value was 53.8 ng/mL, yielding a sensitivity of 79.2% and specificity of 68.4%. By T2, HBP demonstrated excellent discrimination (AUC: 0.919, 95% CI: 0.891–0.948), with a higher optimal cut-off of 112.5 ng/mL (sensitivity: 88.6%, specificity: 82.1%; *p* < 0.001). In contrast, the traditional inflammatory marker CRP exhibited a weaker prognostic predictive ability. At T2, the AUC improved to 0.773 (95% CI: 0.722–0.824), with an optimal cut-off of 24.7 mg/L (sensitivity: 72.3%, specificity: 70.8%; *p* < 0.001). The combination of HBP and CRP significantly outperformed individual biomarkers at T2 (AUC: 0.933, 95% CI: 0.908–0.958; *p* < 0.001). The combined cut-off value for T2 was HBP ≥ 110.3 ng/mL + CRP ≥ 23.1 mg/L, achieving a sensitivity of 91.5% and specificity of 85.3%.

Analysis of HBP kinetics revealed that the relative change from T0 to T2 [(HBP T2 − HBP T0)/HBP T0 × 100%] provided additional prognostic value (Figure 3B). A relative increase ≥108.4% was associated with an AUC of 0.847 (95% CI: 0.798–0.896), sensitivity of 80.9%, and specificity of 78.6% (*p* < 0.001), indicating that monitoring dynamic trends enhances risk stratification.

### Time-dependent ROC curve analysis and AUC values

**Figure 4:**
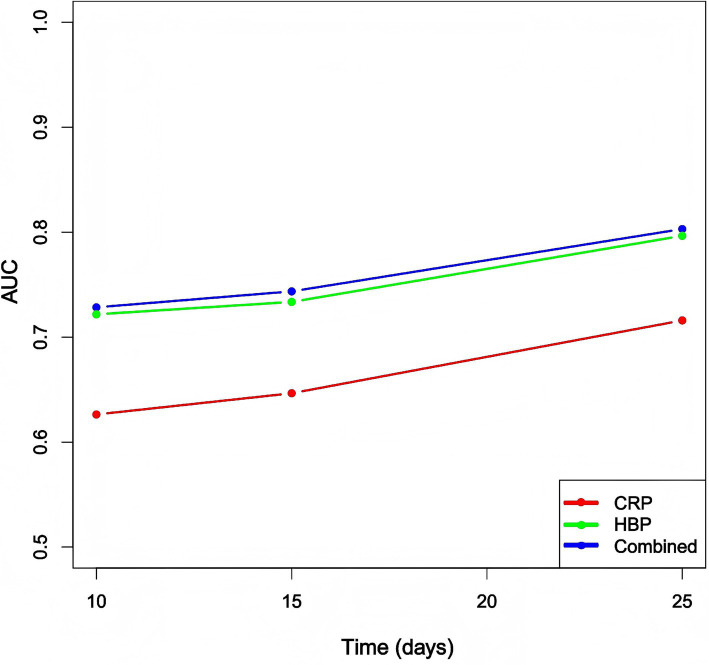
Comparison of time-dependent AUC values for each model at different time points. CRP: C-reactive protein; HBP: Heparin-binding protein.

We calculated the time-dependent area under the curve (AUC) values for the heparin-binding protein (HBP), C-reactive protein (CRP), and the combined model at different time points: Day 10 (D10), Day 15 (D15), and Day 20 (D20). The results are as follows: The AUC values for the HBP model were 0.721, 0.734, and 0.797 at D10, D15, and D20, respectively. The AUC values for the CRP model at the corresponding time points were 0.626, 0.647, and 0.716. The AUC values for the combined model at these time points were 0.728, 0.744, and 0.803 (Figure 4).

**Figure 5:**
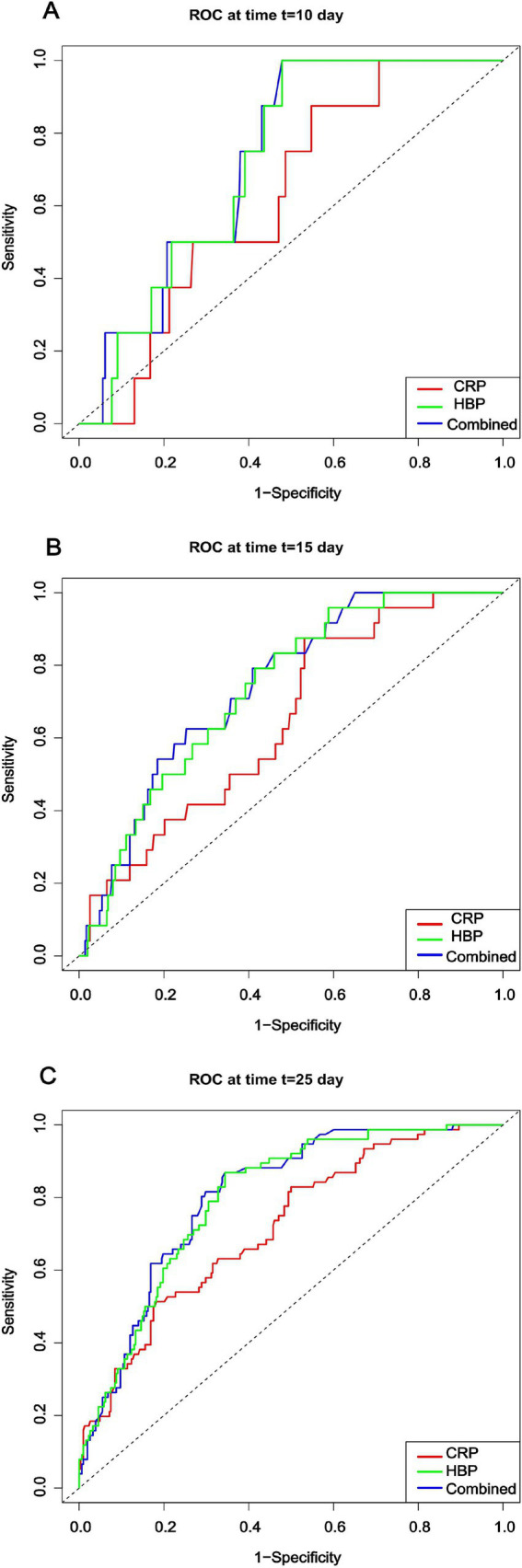
Time-dependent ROC curves for different models at each time point. CRP: C-reactive protein; HBP: Heparin-binding protein.

The plotted time-dependent ROC curves (Figure 5) show that the combined model consistently maintained the highest ROC curve across all time points. Although the ROC curves for the HBP and CRP models intersected, the HBP model generally outperformed the CRP model, suggesting that the combined model exhibited the best discriminative ability throughout the follow-up period. Analysis of the dynamic changes in HBP levels revealed that the AUC values for the HBP model exhibited a noticeable upward trend over time, indicating that dynamic monitoring of HBP is beneficial for capturing disease progression and holds significant value for prognostic assessment. However, compared to the combined model, the predictive capability of the HBP-only model still showed a certain gap, highlighting the necessity for combined testing.

### Cox regression analysis results of HBP dynamic values by subgroup

A time-dependent Cox regression model was employed to analyze the correlation between dynamic levels of heparin-binding protein (HBP) and the 28-day mortality risk in patients. The results were further examined in detail across subgroups based on sex, age, hypertension, diabetes, and infection site. The model’s goodness of fit was assessed using the C-statistic from the Cox proportional hazards analysis, yielding a C-statistic of 0.863 (standard error = 0.017). This indicates that the model demonstrates good discriminative ability in predicting the 28-day mortality risk (*p* < 0.001). Table 2 illustrates that the dynamic levels of HBP were significantly associated with the 28-day mortality risk across all subgroups, further confirming the clinical utility of HBP as a potential prognostic indicator.

**Table 2:** Cox regression analysis results of HBP dynamic values by subgroup.

Subgroup	*n*	Events	HBPT0 coefficient (HR)	HBPT0 *p*-value	HBPT1 coefficient (HR)	HBPT1 *p*-value	HBPT2 coefficient (HR)	HBPT2 *p*-value	HR (95% CI)
Gender									
Male	189	44	−0.0035	0.169	0.0027	0.375	0.0162	<0.001	1.016 (1.010–1.022)
Female	197	36	−0.0136	<0.001	0.0076	0.011	0.0315	<0.001	1.032 (1.021–1.044)
Age group									
65–74 years	47	16	−0.0004	0.917	0.0093	0.179	0.0076	0.032	1.008 (1.001–1.015)
75–85 years	122	12	−0.0117	0.211	0.0064	0.165	0.0314	0.018	1.032 (1.005–1.059)
>85 years	217	52	−0.0039	0.236	0.0006	0.840	0.0209	<0.001	1.021 (1.015–1.028)
Hypertension									
Yes	236	50	−0.0125	<0.001	0.0127	<0.001	0.0225	<0.001	1.023 (1.015–1.031)
No	150	30	0.0015	0.620	−0.0029	0.520	0.0168	<0.001	1.017 (1.011–1.023)
Diabetes									
Yes	248	45	−0.0101	0.002	0.0015	0.597	0.0256	<0.001	1.026 (1.016–1.035)
No	138	35	−0.0008	0.799	0.0100	0.051	0.0100	0.001	1.010 (1.005–1.016)
Infection site									
Pneumonia	94	23	−0.0043	0.185	0.0060	0.170	0.0151	<0.001	1.015 (1.008–1.022)
Abdominal	104	22	−0.0147	0.112	0.0124	0.166	0.0169	0.001	1.017 (1.007–1.027)
Urinary	65	12	−0.0125	0.163	0.0223	0.037	0.0243	0.005	1.024 (1.007–1.042)
Other Sites	123	23	−0.0030	0.470	0.0016	0.712	0.0223	<0.001	1.023 (1.009–1.036)

HBP, Heparin-binding protein; HR, Hazard Ratio; CI, Confidence Interval; T0, On the day of admission; T1, 24 h after admission; T2, On the third day after admission.

## Discussion

The primary findings of this study indicate a significant association between the dynamic changes in heparin-binding protein (HBP) levels and 28-day mortality in elderly patients with sepsis. Specifically, we observed a notable decline in HBP levels over time in the survival group, while HBP levels in the non-survivor group remained consistently elevated. This suggests that dynamic monitoring of HBP may play an important role in prognostic stratification for sepsis in elderly populations. Our results are consistent with previous studies identifying HBP as an effective prognostic biomarker in sepsis patients (20, 37). For instance, Taha et al. (15) noted in their systematic review that static measurements of HBP could predict mortality in sepsis; however, research on its dynamic changes has been relatively scarce (21). Therefore, this study not only addresses this gap but also emphasizes the importance of dynamic monitoring of HBP levels. Nonetheless, our retrospective study design prohibits us from establishing a causal relationship; HBP should be considered a biomarker reflecting disease severity rather than a direct determinant of mortality. It suggests that clinicians should closely monitor changes in HBP levels when assessing sepsis patients to better predict their prognosis and formulate appropriate treatment strategies. However, it should be noted that we did not evaluate whether clinical interventions based on HBP dynamics would influence patient outcomes; thus, findings are limited to prognostic associations only. This finding provides new directions for future research, prompting further exploration of HBP’s potential applications in sepsis management.

While our study demonstrates a strong association between HBP levels—particularly those measured at T2 (the third day after admission)—and 28-day mortality, the practical application of HBP monitoring in clinical decision-making for sepsis requires further clarification. Our data indicate that HBP at T2 offers the highest predictive accuracy; nonetheless, serial measurements also capture dynamic trends that may provide additional prognostic information. Future research is needed to determine whether single-point or serial monitoring yields superior risk stratification and how to optimally incorporate HBP testing into sepsis management pathways.

It is also important to consider how HBP could be integrated with existing clinical protocols, such as the SOFA score or sepsis bundles, to further enhance risk stratification and potentially guide timely treatment escalation. Published studies suggest that adding HBP to current biomarker panels or clinical algorithms may improve prognostic accuracy, but further investigation is required to define such strategies in practice.

Regarding feasibility, current HBP assays are commercially available and can be measured by ELISA or point-of-care testing, typically with a turnaround time of 1–4 h. However, the routine implementation of HBP testing may be limited by cost, availability, differences in laboratory infrastructure, and local practices—factors which vary considerably across healthcare systems. A clearer, evidence-based framework is needed to define how and when HBP measurement can be feasibly performed and interpreted in real-world clinical settings. Cost-effectiveness, impact on workflow, and influence on patient outcomes should all be addressed in future studies to support potential widespread adoption.

Compared to other studies, the dynamic changes in HBP tracked in our study potentially offer superior prognostic value. Previous investigations have explored the integration of HBP with other key biomarkers in sepsis diagnostic models, highlighting HBP’s potential for early identification of sepsis (22). Additionally, studies have shown that plasma levels of HBP significantly rise hours before the onset of hypotension or organ dysfunction in sepsis patients, further validating HBP as a reliable marker for predicting the progression of sepsis to organ failure (23, 24). Another study emphasized the importance of dynamic monitoring of HBP levels in assessing disease progression, indicating that HBP is not merely a static biomarker but a dynamic indicator that changes over time (25). Recent evaluations of HBP’s diagnostic and prognostic value in critically ill sepsis patients further underscore its potential in clinical management (26, 27). While static levels of HBP have some prognostic value in sepsis patients, the impact of its dynamic changes on prognosis has not been thoroughly investigated. Our study utilized linear mixed-effects models to analyze changes in HBP at different time points, providing a more detailed prognostic assessment. This innovative approach captures the temporal trends of HBP levels, offering more precise evidence for clinical decision-making as a risk stratification tool, rather than as an intervention guide and aiding healthcare professionals in managing sepsis. Nevertheless, further prospective studies are needed to assess whether HBP monitoring can actively improve patient management or outcomes.

C-reactive protein (CRP) is a widely used traditional inflammatory marker in sepsis; however, its predictive capability is relatively limited (28, 29). Additionally, procalcitonin (PCT) and interleukin-6 (IL-6) are also commonly utilized biomarkers in sepsis (30, 31). Research indicates that PCT has high specificity in bacterial infections, effectively distinguishing between bacterial and viral infections (32). However, in the early stages of sepsis, its level changes may not be as sensitive as those of HBP. Compared to HBP, the dynamic changes in PCT may lag in certain situations, particularly in elderly patients, where various factors can influence its levels. IL-6, as a pro-inflammatory cytokine, typically shows significantly elevated levels in sepsis; however, due to its elevation in various inflammatory states, its specificity may not be as robust as that of HBP (4, 33). Although CRP, PCT, and IL-6 are all established biomarkers in the diagnosis and management of sepsis, our study was limited by the availability of complete PCT and IL-6 data for all patients in our cohort. Therefore, we could not perform a direct, head-to-head statistical comparison between dynamic HBP, PCT, and IL-6 performance regarding their sensitivity, specificity, and predictive value. Nevertheless, published studies suggest that PCT, as a bacterial infection marker, is widely used for both diagnosis and antibiotic stewardship, while IL-6 is a key inflammatory cytokine with high sensitivity but limited specificity due to its rise in diverse inflammatory conditions. HBP, by contrast, has been reported to increase earlier in the septic process and reflect endothelial activation, providing potentially useful and complementary prognostic information.

Heparin-binding protein (HBP), also known as azurocidin, is mainly stored in neutrophil granules and is rapidly released into circulation in response to infection and inflammatory stimuli. The rise in HBP during sepsis results from neutrophil activation and degranulation, reflecting acute inflammatory responses. Elevated HBP directly enhances endothelial permeability, contributing to vascular leakage and organ dysfunction, which are key features of sepsis pathophysiology (5, 27). Unlike CRP, which is a liver-produced acute phase reactant, and IL-6 or PCT, which have different induction pathways, HBP can be released much earlier in the disease process, directly linked to neutrophil-pathogen interactions. This rapid and event-specific kinetics may make HBP a more sensitive and early marker for sepsis severity and risk stratification than those conventional biomarkers.

This study further explored the combined predictive capabilities of heparin-binding protein (HBP) and C-reactive protein (CRP) in patients with sepsis. The results indicate that the area under the curve (AUC) values for HBP increased progressively at different time points, reflecting an enhancement in its predictive ability over time. This trend sharply contrasts with the performance of CRP, which, despite showing some improvement in predictive capability at T2, still had an AUC value lower than that of HBP. This finding underscores the relatively limited predictive capacity of CRP, particularly in the elderly patient population. Furthermore, our study revealed that the combined use of HBP and CRP at T2 resulted in an AUC of 0.933, significantly surpassing the results obtained from using HBP or CRP alone. Our results demonstrate that the dynamic changes in HBP are closely related to clinical outcomes, especially in elderly patients, further validating HBP as a reliable marker for predicting the progression of sepsis to organ failure. It is important to emphasize that, consistent with the study’s retrospective design, our results reflect associations rather than proof of a direct effect of HBP monitoring on clinical outcomes. Therefore, the dynamic monitoring of both HBP and CRP not only significantly improves the accuracy of prognostic assessments for sepsis patients but also provides clinicians with a more comprehensive tool for patient risk evaluation, potentially aiding in clinical decision-making. Prospective research should further validate these findings and determine whether dynamic monitoring could guide therapeutic interventions.

Elderly patients often exhibit distinct clinical characteristics and outcomes in acute conditions such as sepsis due to physiological decline, multiple comorbidities, and impaired immune function (34, 35). This phenomenon is corroborated by our study, which found a close correlation between heparin-binding protein (HBP) levels and prognosis in elderly patients. Fleischmann et al. (12) noted that the elderly population is more susceptible to sepsis. Our study focused specifically on elderly patients with sepsis to investigate prognostic biomarkers within this high-risk group, rather than susceptibility to sepsis itself. In our cohort of elderly patients, we observed that persistent elevation of HBP was associated with mortality, while survivors exhibited a decline in HBP levels over time. These temporal dynamics are similar to the trends found in pediatric sepsis (36), where decreased HBP concentrations after 72 h were also linked to better prognosis. However, the mechanisms underlying these trends—such as age-related immune differences—were not addressed or confirmed in our study. Thus, further research is necessary to elucidate potential age-dependent differences in HBP kinetics and their clinical significance.

Our results underscore the significance of HBP levels in disease progression among elderly patients, particularly in the management of acute sepsis. HBP not only serves as a potential biomarker but may also become a critical indicator for assessing prognosis. However, whether clinical decisions adjusted according to HBP changes can improve prognosis needs verification in future large-scale, prospective, controlled studies. This finding provides important guidance for clinicians, suggesting that they should pay closer attention to changes in HBP levels when managing elderly patients with sepsis. By doing so, healthcare providers can develop more personalized and effective treatment plans, ultimately improving patient prognosis and quality of life.

It is important to note, however, that our study focused exclusively on elderly patients with sepsis. As a result, the absolute HBP values, kinetic patterns, optimal cut-off points, and diagnostic or prognostic performance (including AUCs) identified in this study reflect the characteristics of this specific population. At present, there is insufficient evidence to determine whether these findings are directly applicable or comparable to non-elderly adult sepsis patients. Age-related differences in immune function, comorbidities, and inflammatory response could potentially influence HBP secretion and metabolism. Accordingly, our results should be interpreted as specific to elderly sepsis patients. Whether the same cut-off values, predictive accuracy, and HBP kinetics are valid in a younger or general adult population remains unknown. Future prospective studies including diverse age groups are needed to assess the comparability and generalizability of HBP as a biomarker across different age cohorts.

The innovation of this study lies in its systematic analysis of the dynamic changes in heparin-binding protein (HBP) levels and their prognostic significance in elderly patients with sepsis. By employing a linear mixed-effects model, we were able to thoroughly assess the effects of time, prognostic groups, and their interactions on HBP levels. This approach not only enhances the accuracy of data analysis but also offers new insights for clinical practice, particularly in the management of elderly sepsis patients. However, the retrospective nature of the study means it cannot determine causality, and findings should be interpreted in this context. Our findings suggest that dynamic monitoring of HBP levels can aid clinicians in the early identification of high-risk patients, thereby potentially optimizing treatment strategies. In contrast to previous studies that primarily focused on the predictive capabilities of static biomarkers, our research emphasizes the importance of dynamic monitoring. We provide evidence that changes in HBP levels can more accurately reflect a patient’s clinical status.

This discovery not only enriches the existing body of research on biomarkers in sepsis but also offers new directions for future studies. It highlights the need to consider the dynamic changes of biomarkers when assessing patients with sepsis to gain a more comprehensive understanding of disease progression and to formulate personalized treatment plans. Through this approach, we aim to advance clinical practice and provide a more scientific and effective basis for managing elderly patients with sepsis.

While this study provides important clinical insights, it also has several limitations. First, the retrospective cohort study design inherently introduces the potential for selection bias and information bias, which may affect the accuracy and reliability of the findings. Additionally, the relatively small sample size limits the generalizability and applicability of the results, potentially impacting their practical implications for clinical practice. Moreover, the dynamic changes in heparin-binding protein (HBP) levels are influenced by various factors, including different types of infections, underlying patient conditions, and the selected treatment strategies. These factors were not adequately controlled in our study, which may introduce some bias in the analysis of HBP changes. Most importantly, due to the observational nature of this research, causality between HBP levels and patient prognosis cannot be inferred. Therefore, future research should emphasize the use of prospective designs, larger sample sizes, and multi-center approaches to comprehensively and systematically validate the findings of this study. Additionally, it is important to explore the influencing factors of HBP’s dynamic changes in depth, aiming to provide a more solid scientific basis for clinical decision-making. Furthermore, a notable limitation is that due to lacking complete PCT and IL-6 data, we were unable to directly compare the prognostic utility of HBP with these well-established biomarkers in our cohort. Future prospective studies including comprehensive biomarker panels are needed to address this gap. Additionally, our study did not assess the operational feasibility, cost, or practical workflow impact of routine HBP measurement in clinical settings, nor did it establish the optimal timing or threshold for interpretation. Further work is needed to address these practical barriers and to evaluate the true utility and implementation of HBP monitoring in routine sepsis care. In addition, it should be considered that HBP levels may be influenced by common treatments in sepsis management. For instance, heparins used for thromboprophylaxis can bind HBP in the bloodstream and potentially alter its measured levels. Furthermore, corticosteroids and certain immunomodulatory agents may affect neutrophil activation and HBP release. Although our study cohort was not stratified by these interventions, such treatment effects could confound HBP interpretation in clinical practice and warrant further dedicated investigation. Clinicians interpreting HBP results should keep these possible influences in mind. Another important limitation is that our study only examined HBP concentrations during the first 3 days after admission. The true onset of sepsis is frequently difficult to determine in clinical practice, particularly among elderly patients, and HBP dynamics may vary over the full course of illness. Due to the retrospective nature and limitations in our dataset, we were unable to analyze HBP kinetics beyond Day 3. Therefore, it remains unclear whether HBP remains elevated, decreases, or exhibits further secondary changes during later stages, such as at 5 or 7 days after admission. Future prospective studies with extended sampling and longer follow-up are needed to elucidate the later-phase kinetics of HBP and clarify its prognostic value throughout the entire course of sepsis.

## Conclusion

In summary, this study highlights the significant association between dynamic monitoring of heparin-binding protein (HBP) levels and 28-day mortality in elderly patients with sepsis. Our findings indicate that survivors exhibited a significant decline in HBP levels, while non-survivors showed persistently elevated levels, suggesting that HBP has potential value as a prognostic biomarker in this population. Furthermore, a predictive model combining HBP with C-reactive protein (CRP) demonstrated improved accuracy for mortality risk assessment, emphasizing the potential utility of multimodal biomarker approaches in clinical practice.

However, it is important to acknowledge that, as a retrospective cohort study, our findings demonstrate only an association rather than a causal relationship between HBP levels and patient outcomes. We did not assess any interventions guided by HBP monitoring, nor can our results determine whether such monitoring would improve clinical outcomes. Therefore, dynamic HBP measurement should currently be considered as an aid for risk stratification and clinical decision-making, rather than a direct means to improve prognosis. Future studies, especially those with larger sample sizes, multicenter involvement, and prospective or interventional designs, are needed to validate our results and to further clarify the clinical utility and potential impact of dynamic HBP monitoring in the management of elderly patients with sepsis.

## Data Availability

The raw data supporting the conclusions of this article will be made available by the authors, without undue reservation.
